# Rare cause of Hemophagocytic Lymphohistiocytosis due to mutation in *PRF1* and *SH2D1A* genes in two children – a case report with a review

**DOI:** 10.1186/s12887-019-1444-4

**Published:** 2019-03-08

**Authors:** Jayesh Sheth, Akash Patel, Raju Shah, Riddhi Bhavsar, Sunil Trivedi, Frenny Sheth

**Affiliations:** 1 FRIGE’s Institute of Human Genetics, FRIGE House, Jodhpur Gam Road, Satellite, Ahmedabad, Gujarat 380015 India; 2 Ankur Institute of Child Health, Behind City Gold Cinema, Ashram Road, Navrangpura, Ahmedabad, Gujarat 380009 India

**Keywords:** Case report, Hemophagocytic Lymphohistiocytosis, HLH, *PRF1*, *SH2D1A*, Next generation sequencing, Carrier status

## Abstract

**Background:**

Hemophagocytic Lymphohistiocytosis (HLH) is a rare, complex, life-threatening hyper-inflammatory condition due to over activation of lymphocytes mediated secretory cytokines in the body. It occurs as a primary HLH due to genetic defect that mostly occurs in the childhood and associated with early neonatal death. Secondary HLH is triggered by secondary to infection and can occur at any age.

**Case presentation:**

The current report presents two cases of HLH. Case 1, three-months-old boy born to second degree consanguineous parents was clinically suspected with HLH. A pathogenic variant in exon 2 of *PRF1* gene [c.386G > C (p.Trp129Ser); FLH-type2] was detected. The parents and the fetus under investigation were shown to be heterozygous carriers, while Case-1 was homozygous for the said variant. Case 2, a one and half-year old male child referred for work-up was born to non-consanguineous young parents. His HLH suspicion was in accordance with HLH-2004 Revised diagnostic guidelines (fulfilling 5/8 criteria). Molecular study revealed hemizygous likely pathogenic variant c.138-3C > G in intron 1 of *SH2D1A* gene. Both the mother and younger sister were confirmed to be the carrier of the same variant.

**Conclusion:**

This study has represented two rare cases of HLH carrying missense variant in *PRF1* and splice site variant in *SH2D1A* gene. Detailed molecular analysis has helped the families with precise genetic counselling and prenatal diagnosis during subsequent pregnancy. It is advocated that male patients presenting with EBV-associated HLH may be screened for XLP that may lead to early diagnosis and therapeutic implication if any.

**Electronic supplementary material:**

The online version of this article (10.1186/s12887-019-1444-4) contains supplementary material, which is available to authorized users.

## Background

Hemophagocytic Lymphohistiocytosis (HLH) is a rare, complex and life-threatening syndrome which is characterized by hyper-inflammation due to over-activation of lymphocytes that releases secretory cytokines in the body [1]. The actual epidemiology of HLH is difficult; however, overall estimated prevalence of HLH in patients younger than 18 years from different ethnic groups is approximately 1 per 100,000 [2]. HLH is classified in two groups, primary or genetic form (P-HLH) where genetic defect is an underlying cause. P-HLH is normally inherited in an autosomal recessive pattern and mostly observed in childhood leading to early death. Secondary or acquired HLH is triggered due to viral infection, autoimmune/rheumatologic, malignant or metabolic conditions and is seen across all age groups [3].

Primary-HLH is further classified in two groups; Familial HLH (FHL) and Lymphoproliferative disorder [4]. Worldwide incidence of FHL is not known. However, extensive studies on FHL are carried out in Sweden and are shown to occur at an estimated incidence of 1 in 50,000 live births [5]. FHL syndromes are sub-classified into FHL-1 through FHL-5, depending upon the gene/s involved and alterations in the functional protein. FHL has an autosomal recessive inheritance and is more commonly seen in the consanguineous families [6, 7]. The prominent feature of hemophagocytosis is seen during early disease course. It very rarely occurs in secondary HLH or might be observed late after the disease has progressed [1].

The diagnosis of primary and/or secondary HLH is carried out – either on clinical presentations or molecular findings or both as suggested by the Histiocyte Society [8].

Nearly 58% FHL cases are reported to harbour mutations in *PRF1* gene (located at 10q22.1) that alters the normal function of perforin [8]; whereas 40–60% of all X-linked lymphoproliferative disorders harbour mutation in SLAM-associated protein (SAP) regulating gene *SH2D1A (*located at Xq25) [9]. One hundred eight disease-causing mutations in the *PRF1* gene and 99 disease causing mutations in the *SH2D1A* gene have been reported so far in the literature [10, 11] including 14 HLH cases from India attributed to mutations in the *PRF1* gene [12–14]. We report here two cases of HLH with the mutation in *PRF1* and *SH2D1A* gene each.

To the best of our knowledge the molecular studies on mutation in *SH2D1A* gene is reported for the first time from India. Both the cases were enrolled for the study as per the institutional ethics committee guidelines in accordance with Helsinki declaration.

## Case presentation

### Case 1

**Fig. 1:**
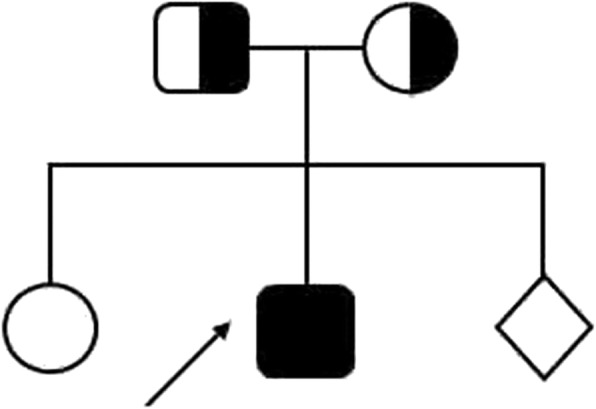
Pedigree chart of Proband (P1)

A young second degree consanguineous parents Fig.1 were referred for genetic counselling during third gravida as their second child (P1) expired at the age of 3 months due to hepatomegaly with pancytopenia. Proband’s elder sister was normal. The proband was born at full term with a birth weight of 3.3 kg. The neonatal period was uneventful. At the age of 3 months, he presented with chief complains of high grade intermittent fever, excessive crying, irritability and reduced oral intake for three days. General examination showed tachycardia and tachypnea. On abdominal examination, the liver was palpable by 4 cms in midclavicular line, firm and nodular in consistency. Haematological examination showed pancytopenia (haemoglobin: 9.5g/dl, Leukopenia: 4660 WBC/cmm, platelets count 55,000/cmm). Biochemical parameters were elevated [serum ALT: 456 U/l, AST: 2348 U/l, ferritin (3994 ng/ml), triglycerides (222 mg/dl), prothrombin time (PT) 22 s and activated partial thromboplastin time (APTT) 50 s]. Serum fibrinogen was decreased (120 mg/dl). Ultrasonography revealed hepatomegaly, mild right pleural effusion, and mild sub-hepatic fluid collection. Bone marrow examination revealed cellular marrow with reactive changes. Based on this, the proband was clinically suspected for HLH.

**Fig. 2:**
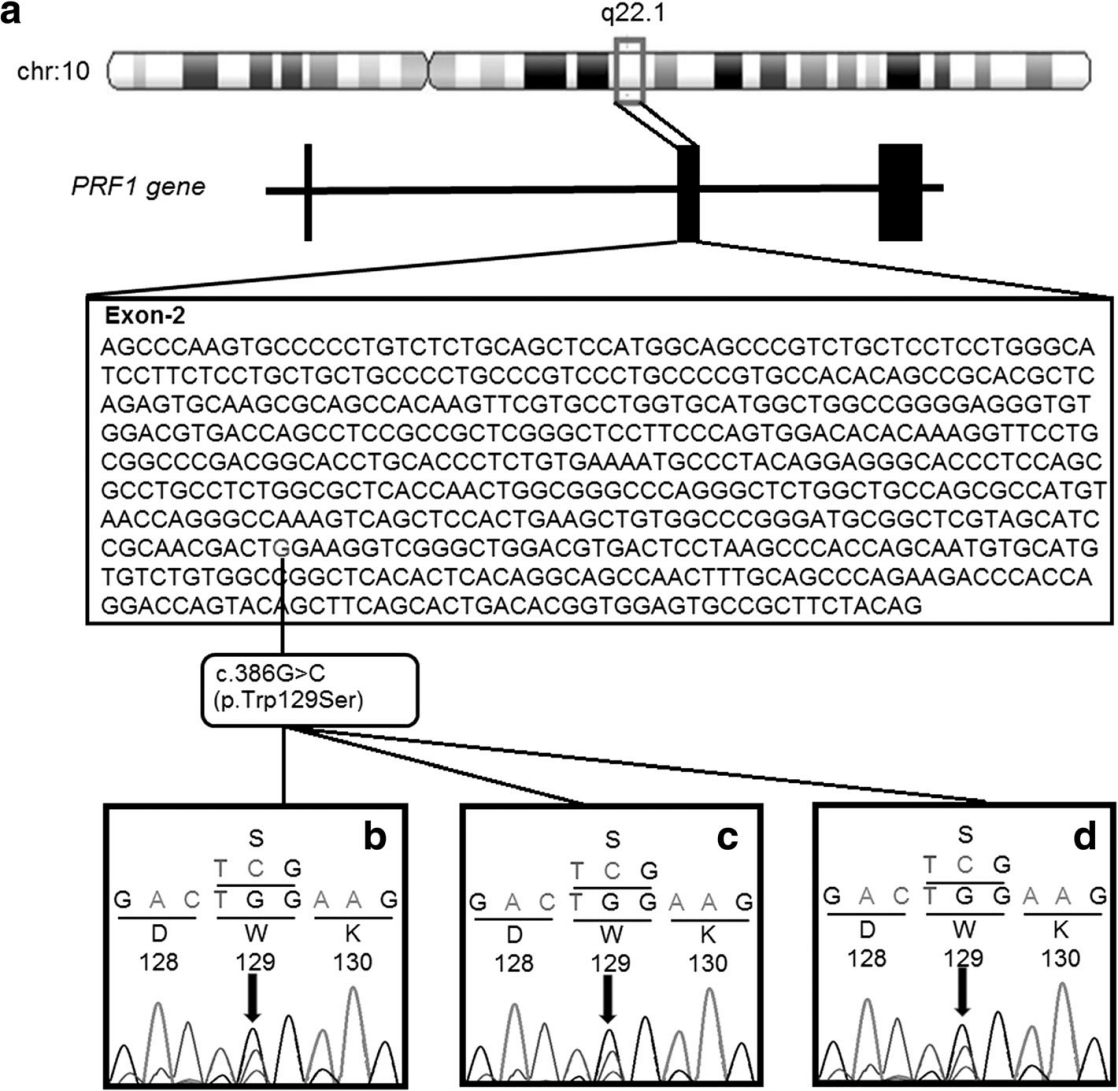
Molecular genetic analysis (**a**) Schematic presentation of *PRF1* gene position on chr10q22.1 and reported homozygous variant c.386G>C/p.Trp129Ser in *PRF1* gene (**b**) Sequence chromatogram of mother (Heterozygous c.386G>C/p.Trp129Ser in *PRF1* gene) **(c)** Sequence chromatogram of Father (Heterozygous c.386G>C/p.Trp129Ser in *PRF1* gene) **(d)** Sequence chromatogram of fetus (Heterozygous c.386G>C/p.Trp129Ser in *PRF1* gene)

The genomic DNA was isolated from peripheral blood [15] and processed for Hemophagocytic Lymphohistiocytosis gene panel on Hiseq-Illumina NGS platform. A homozygous mutant variant c.386G>C (p.Trp129Ser) in exon-2 of *PRF1* gene (OMIM* 170280; NM_005041.5; NP_001076585.1) on chromosome 10q22.1 was detected as shown in Fig. 2. This variant causes substitution of Serine on Tryptophan at codon 129 (ENSP00000398568). In-silico analysis tools like mutation tester, SIFT, Polyphen2 predicted the damaging effect of the said variant. This variant is not reported in the 1000 genome database and found to be damaging (rs768849283). Bidirectional Sanger sequencing further confirmed parents to be heterozygous for the same variant [c.386G > C (p.Trp129Ser) in exon 2 of *PRF1* gene] [Additional file 1]. All these investigations confirmed the autosomal recessive inheritance of FHL2. Prenatal diagnosis from chorionic villus (CVS) was carried out at 12 weeks of gestation which has revealed heterozygous status for c.386G>C variant in exon 2 of *PRF1* gene. A normal carrier child was delivered at full term normal delivery.

### Case-2

**Fig. 3:**
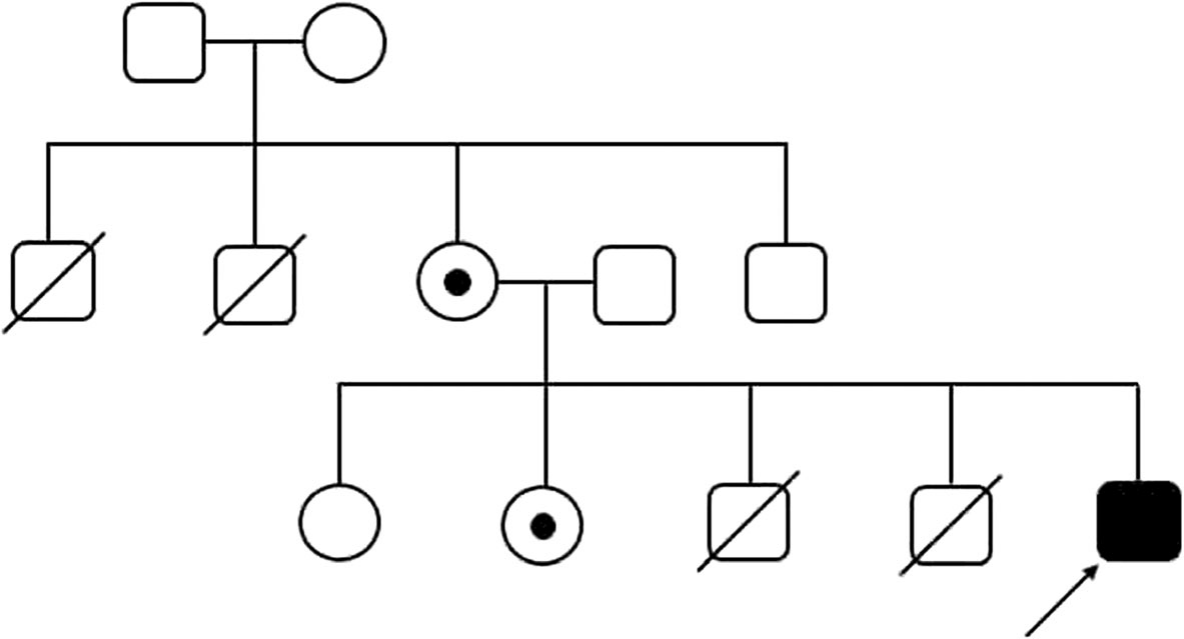
Pedigree chart of Proband (P2)

The male child (P2; 5th gravida) was born at full-term with a birth weight of 2.25 kg. The postnatal period was uneventful. He was born of non-consanguineous young parents Fig. 3. Earlier, the eldest brother of the proband died at an age of 4 days due to prematurity. The second brother died at the age of 3 years with similar complains as of the index case and had two healthy elder normal sisters (aged 14 years and 11 years). Both the brothers of proband’s mother died earlier at an age of 3 years due to high ALT (330 IU/ml) and lactic acid levels. The proband was referred with the chief complain of persistent fever (15 days’ duration) at the age of 16 months and had one episode of focal seizure with twitching over the face (which lasted for 2–3 min) that subsided on its own within 3 days. At the age of 17 months, the proband was admitted to the hospital due to viral illness. The physical examination revealed a mild oedematous face. Upon abdominal examination, the liver was palpable by 3 cm in midclavicular line, firm and nodular in consistency. The spleen was 2+ cm palpable towards umbilicus, firm and nodular in consistency. There was no facial dysmorphism observed.

**Fig. 4:**
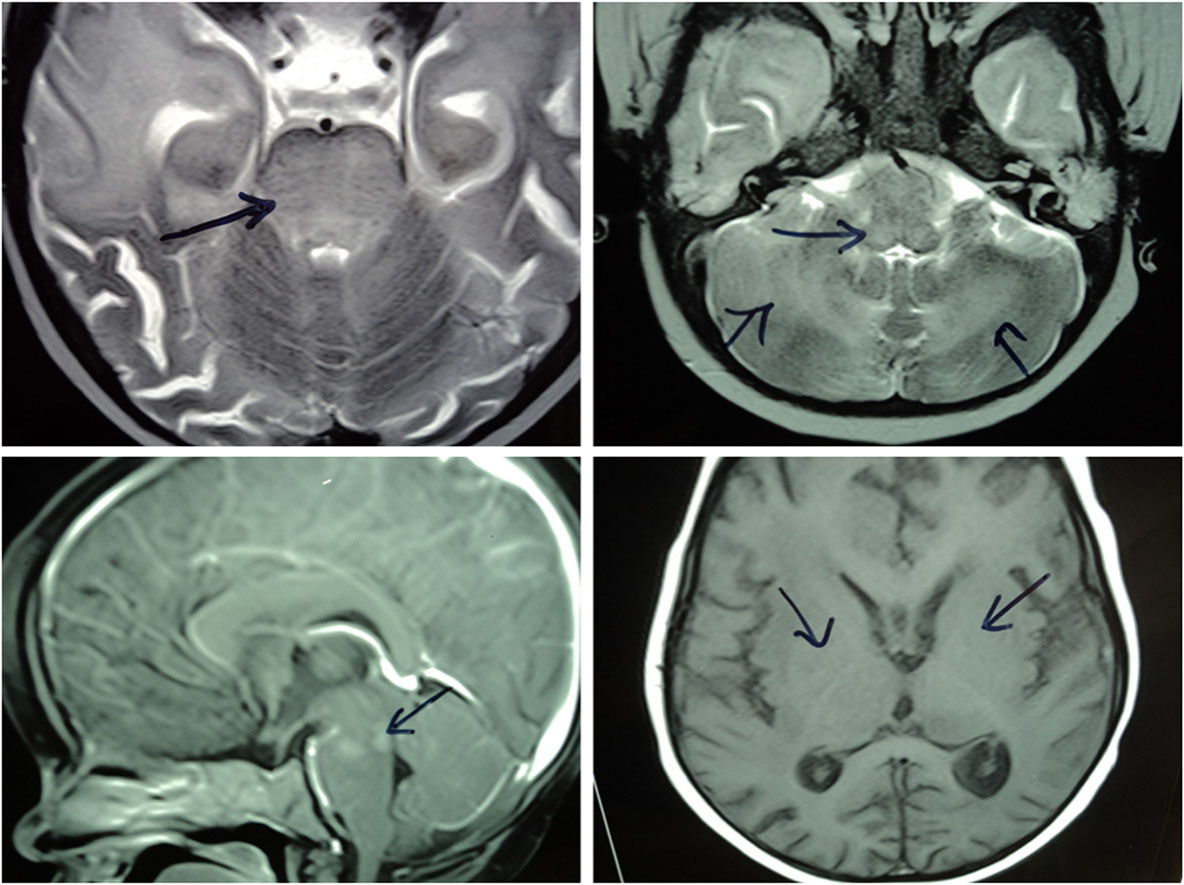
MRI Image of proband (P2). Meningoencephalitis with post-infective extensive demyelination is seen

Haematological investigation revealed peripheral blood with pancytopenia (haemoglobin: 6.9 g/dl, Leukopenia: WBC 2570/cmm, decreased platelets count 3000/cmm). Biochemical parameters were elevated [serum ALT: 300 U/l, prothrombin time (PT) 20 s and activated partial thromboplastin time (aPTT) 58 s]. Cerebrospinal fluid examination was performed due to the history of fever and convulsion and was suggestive of aseptic Meningitis as mentioned in the clinical note. EBV was tested positive by serologic testing. Bone marrow examination revealed cellular marrow aspirate with adequate erythropoiesis, myeloid hyperplasia, toxic changes, and a moderate increase in reticulum fibers, mild eosinophilia and adequate megakaryocyte suggestive of reactive marrow changes. However, there was an evidence of hemophagocytosis on trephine bone marrow biopsy. Ultrasonography revealed mild splenomegaly, moderate hepatomegaly and minimal free fluid in the peritoneal cavity; kidneys, pancreas, gall bladder, and para-aortic region were unremarkable. MRI of the brain was suggestive of meningoencephalitis with post-infective extensive demyelination in all lobes (fronto-temporal-parito-occipital), bilateral involvement of periventricular white matter, basal ganglia, thalamus, brain stem, and cerebellar hemisphere; no fresh infarct, Space-Occupying Lesion (SOL), haemorrhage, a malformation in brain parenchyma Fig. 4. Biochemical studies were performed that ruled out Gaucher and Niemen-Pick–A disease. β-Glucosidase, plasma Chitotriosidase, Acid Sphingomyelinase were also normal. In view of pyrexia of unknown origin (PUO), hepatosplenomegaly, pancytopenia, hypertriglyceridemia, high ferritin and hemophagocytosis, HLH was suspected and clinically confirmed in accordance with Henter-2004 revised diagnostic guidelines (5/8 criteria). The proband died at the age of 19 months due to meningoencephalitis.

**Fig. 5:**
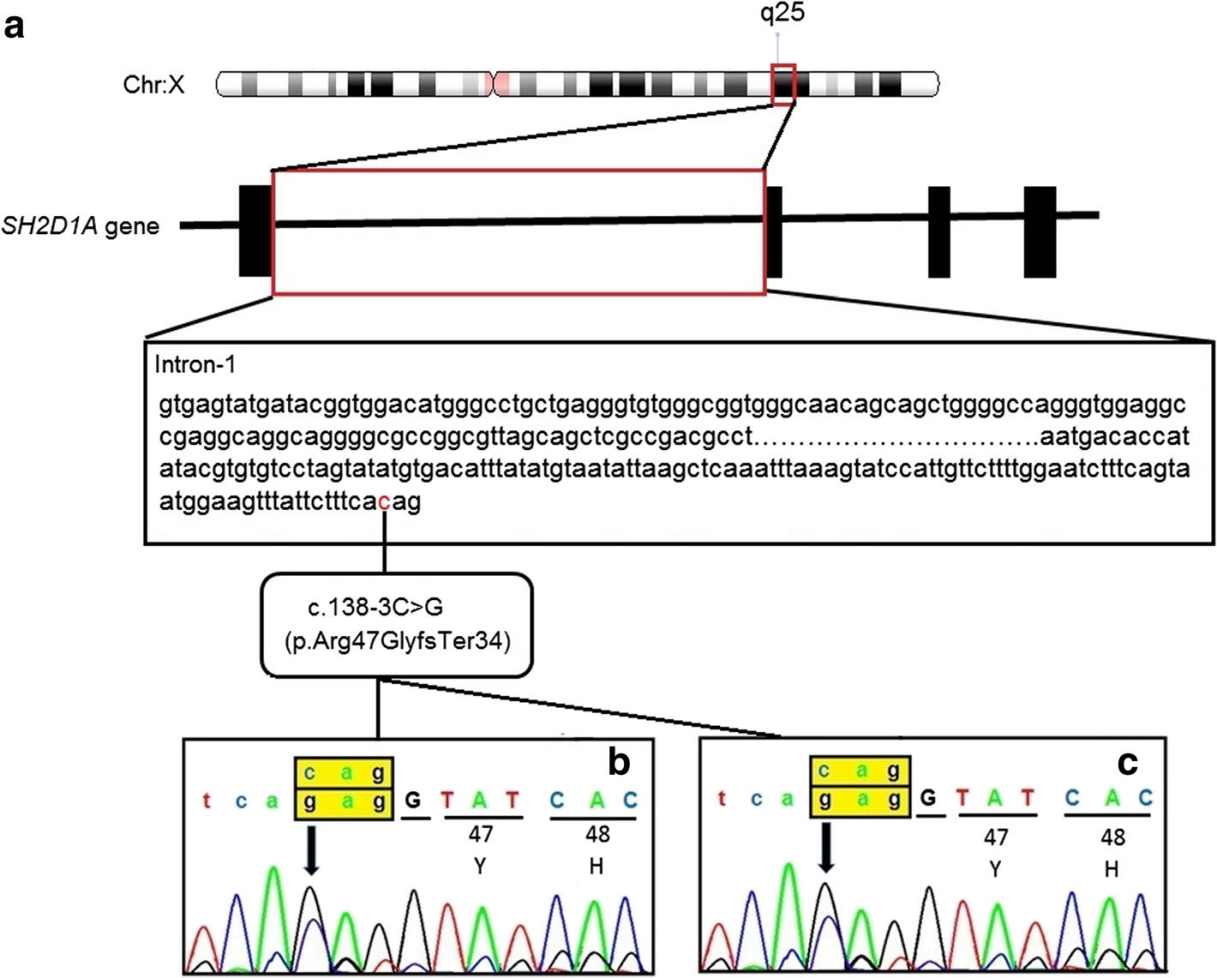
Molecular genetic analysis (**a**) Schematic presentation of *SH2D1A* gene position on chrXq25 and reported variant c.138-3C>G/p.Arg47GlyfsTer34 in Intron-1 of *SH2D1A* gene. (**b**) Sequence chromatogram of mother (Heterozygous c.138-3C>G/p.Arg47GlyfsTer34 in Intron-1 of *SH2D1A* gene. (**c**) Sequence chromatogram of younger sister (Heterozygous c.138-3C>G/p.Arg47GlyfsTer34 in Intron-1 of *SH2D1A* gene

Further confirmation of the diagnosis was made by isolation of genomic DNA from peripheral blood [15] that was subjected to clinical exome sequencing on Miseq-Illumina NGS platform which has identified a hemizygous likely pathogenic variant c.138-3C>G in the intron-1 of *SH2D1A* gene (OMIM*300490; NP_002342.1; NM_002351.4; Fig. 5). In-silico prediction tool (ASSP) further support the effect of this variant affect the splice site of exon-2 and causes frameshift termination (p.Arg47GlyfsTer34) of the protein (80 amino acid residues instead of 128 amino acid residues). Mutation Taster suggested the variant to be disease-causing. This variant was not found either in ExAC or 1000 Genome Databases. Bidirectional Sanger sequencing was performed to validate its presence (Additional file 1). Mother and the younger sister of the proband were investigated and found to be heterozygous for c.138-3C>G mutation in intron-1 of *SH2D1A* gene.

## Discussion and conclusion

Literature survey for mutations in exon-2 of *PRF1* and intron-1 of *SH2D1A* genes are presented in Additional file 2. PRF gene mutations (*N* = 33/108; Additional file 2) span over multiple ethnicities [10]. They are mostly observed in Asian and European population and less commonly seen in American and other populations [10, 11]. Nonsense mutation (p.Trp34Ter) is mostly seen in Turkish population [16, 17], frameshift mutation (c.1090_1091delCT and c.50delT) is more commonly observed in Japanese and African-American population [17, 18] whereas missense mutation (p.Ala91Val and p.Trp129Ser) is seen in Italian and Indian population respectively [12, 13, 19]. Most of the missense mutations commonly seen in HLH are observed under the umbrella of *PRF1* gene. Nonetheless, such ethnic predilection is not reported for *SH2D1A* gene mutation [11].

In our study, proband P1 has shown to have aforesaid disease-causing mutation (p.Trp129Ser) in *PRF1* gene. This gene encodes for perforin protein, located at 10q22.1 region and is obligatory for granule release mechanism of Natural Killer cells (NK-cell) and cytotoxic T lymphocytes (CTL) by forming pores into plasma membrane of the target cells and for passive diffusion of granzyme B for an apoptotic event [13]. This protein is expressed up to 0–3% on CD8+ cells in the children from birth till 1 year of age whereas it is expressed on CD56+ cells from 1 to 30% in lymphocytes from 1 year to 15 years of age [18]. In our case P1, missense mutation p.Trp129Ser in *PRF1* gene is likely to have caused a gain in glycosylation site of protein structure that result in loss of the binding stability with intracellular calcium ions [21]. This renders perforin protein to become non-functional through changes in conformation leading to defective NK cell activity which has been observed in the majority of FHL patients [12–22]. Though present study lacks the functional evidence of defective NK cell activity, Schneider et al. have classified FHL patients into four sub-types based on the level of NK cell activities [23]. On the other hand, none of the cases with homozygous nonsense mutation in *PRF1* gene have shown the evidence of different NK cell activity irrespective of the FHL sub-types. This indicates that *PRF1* mutation is likely to play an important role in the persistence of deficient NK cell activity in FHL cases [24].

Katano et al. had demonstrated accumulation of an uncleared precursor form of perforin and absence of an active mature form of perforin in patients with *PRF1* missense mutation [25]. This indicates that missense mutation of *PRF1* gene causes conformational changes in the protein and inhibits the proteolytic cleavage of perforin precursor. The immune competence of patients with FHL depends on functional analysis of antigen-specific cytotoxic T-lymphocytes (CTLs). Cytotoxic formation in patient with missense mutation in *PRF1* gene remains intact than those having nonsense mutation [24]. Nonetheless, functional analysis of antigen-specific CTLs may yield better insight into the immune competence of patients.

In proband P2, disease-causing hemizygous variant (c.138-3C>G) in intron-1 of *SH2D1A* gene was detected. The same variant was reported earlier by Sayos et al. in a 6-year-old Italian boy [26]. This gene encodes SAP protein, located on Xq25 position [9]. It has a role as a key regulator in normal immune function of all three; NK-T (Natural killer T) cells, T-cells and B-cells. Increased SAP expression is seen in thymus-derived lymphocytes. A SLAM-SAP interaction initiates the lymphocyte activation [26, 27]. Variant c.138-3C>G affects the splice site of exon-2, causing frameshift termination of SAP and leads to dysfunctional form. This alters the SH2 domain that plays a crucial role in binding with SLAM molecule. Thus, dysfunctional SAP protein induces the signal transduction of T-lymphocytes and display defects in their regulation. This defect is likely to cause the impaired production of interferon-gamma by defective Helper T-cell (CD4+ T-cell) leading to an increase in viral replication [26].

A positive Epstein Barr virus test in the proband point towards a failure of immune system in protecting against EBV infection, due to an elimination of EBV-infected B lymphocytes by defective Helper T cells [28] which is likely to be due to defect in NK cell activation and NK cell-mediated cytotoxicity. The model proposed by Parolini et al. [29] suggested that altered function of a 2B4 molecule (SLAM family member) affects the ability to control EBV infection in the body. The authors also showed that in the absence of SAP/2B4-mediated recognition, CD48 play a crucial role in the inhibition of NK cell-mediated killing of EBV-infected B-cell. Benoit et al. [30] also suggested that SAP/2B4 signal alteration leads to immune dysfunction. Immune system failure in the killing of EBV-Infected cells leads to prolonged activation of cytotoxic cells and results in cytokine secretion which activates macrophages. It decreases blood cell count and haemoglobin level which leads to hemophagocytosis in bone marrow and deranged liver functions which has been observed in the case under report. This indicates that the gene variant affects the immune function but after the viral infection, XLP becomes life-threatening as seen in the present case.

XLP1 patients with splice site variants are reported to have two years average median age of onset and predicted survival of 3 years [31] which is in accordance with our observation. This indicates that lack of SAP resulting as a consequence of variants leads to a severe activation of lymphocytes and have a profound effect on the patients. It was also noted that XLP patients who have a nonsense and deletion mutations have a decreased SAP whereas missense and splice site mutations have absent or decreased SAP expression [9].

The patients who are either not treated with specific therapies or undergoing transplantation die mostly due to severe pancytopenia that deregulates immune function. Moreover, it is reported that with fungal, bacterial or viral infection the survival of patients without treatment is < 2 months [32] as seen in both these cases.

In conclusion, our study reports mutation in *PRF1* and *SH2D1A* gene respectively that are known to be associated with HLH. Detailed molecular analysis has helped the families with precise genetic counselling and prenatal diagnosis during subsequent pregnancy. It is also recommended that male patients presenting with EBV-associated HLH need to be screened for XLP for early diagnosis and therapeutic implication if any.

## Additional files

Additional file 1: Protocol for Sanger sequencing. (DOCX 14 kb)

Additional file 2: Genotype and phenotype variation in some reported cases of *SH2D1A* and *PRF1* genes. (DOCX 27 kb)

## Abbreviations

aPTT: Activated partial thromboplastin time
CD: Cluster of Differentiation
CNS: Central nervous system
CSF: Cerebrospinal fluid
EBV: Epstein - Barr virus
ExAC: The Exome Aggregation Consortium
FHL: Familial Hemophagocytic Lymphohistiocytosis
HLH: Hemophagocytic Lymphohistiocytosis
IL2Rα: Interleukin 2 receptor α
MRI: Magnetic resonance imaging
NGS: Next-Generation sequencing
NK cell: Natural killer cell
NK-T cell: Natural killer-T cell
OMIM: Online mendelian inheritance in men
*PRF1*: Perforin1
SAP: SLAM-associated protein
SGOT: Serum glutamic oxaloacetic transaminase
SGPT: Serum glutamic pyruvic transaminase
*SH2D1A*: SH2 Domain 1A
SIFT: Scale-Invariant feature transform
SLAM: Signaling lymphocyte activation molecule
SOL: Space-Occupying Lesion
WBC: White blood cells

## References

[1] George MR (2014). Hemophagocytic lymphohistiocytosis: review of etiologies and management. Journal of Blood Medicine.

[2] Niece JA, Rogers ZR, Ahmad N, Langevin AM, McClain KL (2010). Hemophagocytic lymphohistiocytosis in Texas: observations on ethnicity and race. Pediatr Blood Cancer.

[3] Madkaikar M, Shabrish S, Desai M (2016). Current updates on classification, diagnosis and treatment of Hemophagocytic Lymphohistiocytosis (HLH). Indian J Pediatr.

[4] Waleed A, Bousfiha A, Casonova JL, Chatila T, Conley ME, Cunningham-Rundles C (2014). Primary immunodeficiency diseases: an update on the classification from the International Union of Immunological Societies Expert Committee for primary immunodeficiency. Front Immunol.

[5] Henter JI, Elinder G, Soder O, Ost A (1991). Incidence in Sweden and clinical features of familial hemophagocytic lymphohistiocytosis. Acta Paediatr Scand.

[6] Mehta RS, Smith ER (2013). Hemophagocytic lymphohistiocytosis (HLH): a review of literature. Med Oncol.

[7] Ericson KG, Fadeel B, Arndor SN, Soderhall C, Samualsson A, Janka G (2001). Spectrum of perforin gene mutations in familial Hemophagocytic Lymphohistiocytosis. Am J Hum Genet.

[8] Henter JI, Horne A, Arico M, Egeler RM, Filipovich AH, Imasuku S (2006). HLH-2004: diagnostics and therapeutic guidelines for Hemophagocytic Lymphohistiocytosis. Pediatr Blood Cancer.

[9] Filipovich AH, Zhang K, Snow AL, Marsh RA (2010). X-linked lymphoproliferative syndromes: brothers or distant cousins?. Blood..

[10] HGMD Database. http://www.hgmd.cf.ac.uk/ac/gene.php?gene=PRF1 Accessed 14 sept 2018.

[11] HGMD Database. http://www.hgmd.cf.ac.uk/ac/gene.php?gene=SH2D1A Accessed 14 sept 2018.

[12] Mhatre S, Madkaikar M, Jijina F, Ghosh K (2014). Unusual clinical presentation of familial Hemophagocytic Lymphohistiocytosis Type-2. JPediatrHematol Oncol.

[13] Mhatre S, Madkaikar M, Desai M, Ghosh K (2015). Spectrum of perforin gene mutation in familial lymphohistiocytosis (FHL) patients in India. Blood cells Mol Disease.

[14] Madkaikar M, Gupta M, Dixit A, Patil V (2017). Predominant neurologic manifestations seen in a patient with a Biallelic Perforin1 mutation (PRF1; p.R225W). J Pediatr Hematol Oncol.

[15] Miller SA, Dykes DD, Polesky HF (1988). A simple salting out procedure for extracting DNA from human nucleated cells. Nucleic Acids Res.

[16] Zur Stadt BK, Kolberg K, Schneppenheim R, Kabisch H, Janka G (2006). Mutation Spectrum in children with primary Hemophagocytic Lymphohistiocytosis: molecular and functional analyses of PRF1, UNC13D,STX11, and RAB27A. Hum Mutat.

[17] Omar AN, Gursoy A, Gurgey A, Keskin O (2013). Structural and functional analysis of perforin mutations in association with clinical data of familial hemophagocytic lymphohistiocytosis type 2 (FHL2) patients. Protein Sci.

[18] Ueda I, Morimoto A, Inaba T, Yagi T, Hibi S, Sugimoto T (2003). Characteristic perforin gene mutations of hemophagocytic lymphohistiocytosis patients in Japan. Br J Haematol.

[19] Clementi R, Emmi L, Maccario R, Liotta F, Moretta L, Danesino C (2002). Adult onset and atypical presentation of hemophagocytic lymphohistiocytosis in sibling carrying PRF1mutation. Blood..

[20] Chia J, Thia K, Brennan AJ, Little M, Williams B, Lopez JA (2012). Fatal immune dysregulation due to gain of glycosylation mutation in lymphocyte perforin. Blood..

[21] Kogawa K, Lee SM, Villanueva J, Marmer D, Sumegi J, Filipovich AH (2002). Perforin expression in cytotoxic lymphocytes from patients with hemophagocytic lymphohistiocytosis and their family members. Blood..

[22] Feldmann J, Deist F (2002). Ouache´e-Chardin M, Certain S, Sarah A, Quartier P, et al. Functional consequences of perforin gene mutations in 22 patients with familial haemophagocytic lymphohistiocytosis. Br J Haematol.

[23] Schneider EM, Lorenz I, Muller-Rosenberger M, Steinbach G, Kron M, Janka-Schaub GE (2002). Hemophagocytic lymphohistiocytosis (HLH) is associated with deficiencies of cellular cytolysis but normal expression of apoptosis related transcripts. Blood..

[24] Ishii E, Ueda I, Shirakawa R, Yamamoto K, Horiuchi H, Ohga S (2005). Genetic subtype of familial hemophagocytic lymphohistiocytosis: correlations with clinical features and cytotoxic T lymphocytes/natural killer cell function. Blood..

[25] Katano H, Ali MA, Patera AC, Catalafamo M, Jaffe ES, Kimura H (2003). Chronic active Epstein-Barr virus infection associated with mutations in perforin that impair its maturation. Blood..

[26] Sayos J, Wu C, Morra M, Wang N, Zhang X, Allen D (1998). The X-linked lymphoprolifertaive-disease gene product SAP regulates signal induced through the co-receptor SLAM. Nature..

[27] Nichols KE, Ma CS, Cannons JL, Schwartzberg PL, Tangye SG (2005). Molecular and cellular pathogenesis of X-linked lymphoproliferative disease. Immunol Rev.

[28] Sumegi J, Seemayer TA, Huang D, Davis JR, Morra M, Gross TG, et al. A spectrum of mutations in SH2D1A that causes X-linked lymphoproliferative disease and other Epstein-Barr virus-associated illnesses. Leuk Lymphoma. 2002;43:1189-01. 10.1080/10428190290026240 12152986

[29] Parolini S, Bottino C, Falco M, Augugliaro R, Giliani S, Franceschini R (2000). X-linked lymphoprolifertaive disease: 2B4 molecules displaying inhibitory rather than activating function are reponsible for the inability of natural killer cells to kill Epstein-Barr virus infected cells. J Exp Med.

[30] Benoit L., Wang X., Pabst H.F., Dutz, J, Tan R. Defective NK cell activation in X-linked lymphoproliferative disease. J Immunol. 2000;165:3549–53. 10.4049/jimmunol.165.7.3549 11034354

[31] Sumegi J, Huang D, Lanyi A, Davis JD, Seemayer TA, Maeda A, et al. Correlation of mutation of SH2D1A gene and Epstein-Barr virus infection with clinical phenotypes and outcome in X-linked lymphoproliferative disease. Blood. 2000;96:3118-25. 11049992

[32] Janka GE (1983). Familial hemophagocytic lymphohistiocytosis. Eur J Pediatr.

