# Supplementary material for: Rare cause of Hemophagocytic Lymphohistiocytosis due to mutation in PRF1 and SH2D1A genes in two children – a case report with a review
Source: BMC Pediatr. 2019 Mar 8;19:73. doi: 10.1186/s12887-019-1444-4 (PMC6407181; doi:10.1186/s12887-019-1444-4)
Supplement: Supplementary file 1 — Protocol for Sanger sequencing. (DOCX 14 kb) [file 12887_2019_1444_MOESM1_ESM.docx]

**Supplementary file-1**

**Sanger sequencing (Confirmative Test)**

In Case-1, Fetus and both the parents DNA samples with a concentration of 150 ng/µL were amplified using Thermal Cycler-2720 (Applied Biosystems). Exon 2 of *PRF1* gene was amplified using a forward primer sequence 5'TGGACACACAAAGGTTCCTG3' and reverse primer sequence 5'TCACCTGTAGAAGCGGCACT3'. A total of 30 cycles of Polymerase Chain Reactions (PCR) were run with initial denaturation (94°C; 5 minutes), denaturation (94°C; 30 seconds), annealing (63°C; 45 seconds), elongation (72°C; 1 minute) and final elongation (72°C; 7 minutes). The amplifications of the PCR products were confirmed by 2.5% agarose gel electrophoresis. Using these PCR products, sequencing was carried out on the SeqStudio genetic analyzer.

In Case-2, proband mother and younger sister’s both DNA samples with a concentration of 150 ng/µL were amplified using Thermal Cycler-2720 (Applied Biosystems). Intron-1 of SH2D1A gene was amplified using a forward primer sequence 5'CTCACTGGAAACTGTGGTTGG3' and reverse primer sequence 5'CTCCTTGACACCCCCAGAA3'. A total of 35 cycles of Polymerase Chain Reactions (PCR) were run with initial denaturation (94°C; 5 minutes), denaturation (94°C; 30 seconds), annealing (63°C; 45 seconds), elongation (72°C; 1 minute) and final elongation (72°C; 7 minutes). The amplifications of the PCR products were confirmed by 2.5% agarose gel electrophoresis. Using these PCR products, sequencing was carried out on the SeqStudio genetic analyzer.
