# Supplementary material for: Rare cause of Hemophagocytic Lymphohistiocytosis due to mutation in PRF1 and SH2D1A genes in two children – a case report with a review
Source: BMC Pediatr. 2019 Mar 8;19:73. doi: 10.1186/s12887-019-1444-4 (PMC6407181; doi:10.1186/s12887-019-1444-4)
Supplement: Supplementary file 2 — Genotype and phenotype variation in some reported cases of SH2D1A and PRF1 genes. (DOCX 27 kb) [file 12887_2019_1444_MOESM2_ESM.docx]

Supplementary File-2:

Few reported disease causing variants of *PRF1 and SH2D1A* gene variants similar to current study.

| Gene | Exon/  Intron/ | Variation | Age/Sex | Ethnicity/  Country of Origin | Clinical Indication | Author |
| --- | --- | --- | --- | --- | --- | --- |
| *PRF1* | Exon-2 | c.50DelT (p.Leu17fsTer) | N/A* | African- American | Hepatomegaly, splenomegaly, neutropenia, and hypofibrinogenaemia, hypertriglyceridemia, fever, and thrombocytopenia | Stepp SE. et al., 1999 [1] |
|  | Exon-2 | c.190C>T (p.Gln64Ter) | N/A | N/A | Hepatomegaly, splenomegaly, neutropenia, and hypofibrinogenaemia, hypertriglyceridemia, fever, and thrombocytopenia | Stepp SE. et al., 1999 [1] |
|  | Exon-2 | c.148G>A (p.Val50Met) | 4m/M | Turkey | Hypertriglyceridemia, hypofibrinogenemia, Fever, Splenomegaly, cytopenia, hemophagocytosis, | Ericson G. et al., 2001 [2] |
|  | Exon-2 | c.283T>C (p.Trp94Arg) | 2m/M | Italy | Fever, Hepatomegaly, skin rash, abnormal Hb level and platelet count, neutropenia, hypertriglyceridaemia or hypofibrinogenaemia, and CSF pleocytosis | Clementi R. et al.,2001 [3] |
|  | Exon-2 | c.116C>A (p.Pro39His) | 10y/F | N/A | Defective NK cell and T-cell activity, hepatomegaly, splenomegaly, neutropenia, and hypofibrinogenaemia. lymphopenia, hypertriglyceridaemia, central nervous system involvement, fever, anaemia, and thrombocytopenia | Kogawa K. et al., 2002 [4] |
|  | Exon-2 | c.133G>A (p.Gly45Arg) | 3y/F | Hispanic | Defective NK cell and T-cell activity h hepatomegaly, splenomegaly, neutropenia, and hypofibrinogenaemia. Lymphopenia, hypertriglyceridaemia, central nervous system involvement, fever, anaemia, and thrombocytopenia | Kogawa K. et al., 2002 [4] |
|  | Exon-2 | c.160C>T (p.Arg54Cys) | 3y/F | Hispanic | Defective NK cell and T-cell activity, hepatomegaly, splenomegaly, neutropenia, and hypofibrinogenaemia. lymphopenia, hypertriglyceridaemia, central nervous system involvement, fever, anaemia, and thrombocytopenia | Kogawa K. et al., 2002 [4] |
|  | Exon-2 | c.445G>A (p.Gly149Ser) | 10y/F | N/A | Defective NK cell and T-cell activity, hepatomegaly, splenomegaly, neutropenia, andhypofibrinogenaemia. Lymphopenia, hypertriglyceridaemia, CNS involvement, fever, anaemia, and thrombocytopenia | Kogawa K. et al., 2002 [4~~]~~ |
|  | Exon-2 | c.272C>T (p.Ala91Val) | 27y/F | Italy | Fever, weight loss, weakness, and hepatosplenomegaly,pancytopenia, hypertriglyceridemia, hypofibrinogenemia and highlevels of ferritin | Clementi R. et al.,2002[5] |
|  | Exon-2 | C.3G>A (p.Met1Ala) | 1y/N/A | Lebanon | Fever, hepatosplenomegaly, pancytopenia, liver dysfunction, high ferritin and triglycerides level | Feldmann J. et al., 2002 [6] |
|  | Exon-2 | c.1A>G (p.Met1Gly) | 11y/F | Japan | CNS involvement, defective NK cell activity, | Ueda I. et al., 2003 [7] |
|  | Exon-2 | c.265C>A (p.Pro89Thr) | N/A | Oman | hypoalbuminemia, hyperferritinemia, and hypertriglyceridemia | Al-Lamki Z. et al., 2003 [8] |
|  | Exon-2 | c.208G>T (p.Asp70Tyr) | 8y/M | N/A | Hepatomegaly, splenomegaly, neutropenia, hypofibrinogenemia. Lymphopenia, hypertriglyceridemia, central nervous system involvement, fever, anemia, and thrombocytopenia | Molleran L. et al., 2004  [9] |
|  | Exon-2 | c.217T>C (p.Cys73Arg) | 4m/M | African American | Hepatomegaly, splenomegaly, neutropenia, hypofibrinogenemia. Lymphopenia, hypertriglyceridemia, central nervous system involvement, fever, anemia, and thrombocytopenia | Molleran L. et al., 2004 [9] |
|  | Exon-2 | c.449C>A (p.Ser150Ter) | 5y/F | N/A | Hepatomegaly, splenomegaly, neutropenia, and hypofibrinogenaemia. Lymphopenia, hypertriglyceridaemia, central nervous system involvement, fever, anaemia, and thrombocytopenia | Molleran L. et al., 2004 [9] |
|  | Exon-2 | c.459T>G (p.Phe157Val) | 2m/F | N/A | Hepatomegaly, splenomegaly, neutropenia, and hypofibrinogenaemia. lymphopenia, hypertriglyceridaemia, central nervous system involvement, fever, anaemia, and thrombocytopenia | Molleran L. et al., 2004 [9] |
|  | Exon-2 | c.112G>A (p.Val38Met) | 6y/N/A | Morocco | Low fibrinogen level, high ferritin and triglyceride level, high conc. of T-cell and NK cell, hemophagocytosis in bone marrow, CSF and lymphnodes | Zur Stadt et al.,2006 [10] |
|  | Exon-2 | c.185_195del11 (p.Asp62fsX12) | 2m/N/A | Germany | Fever, hepatosplenomegaly, neutropenia, thrombocytopenia, high ferritin and triglycerides level | Zur Stadt et al.,2006 [10] |
|  | Exon-2 | c.508A>C (p.Ser170Arg) | 18m/M | America | Elevated triglyceride level and ferritin level ,elevated serum lactate dehydrogenase level , mild pancytopenia, hematocrit, elevated aspartate aminotransferase level , lethargic, hyper-reflexia and hypertonicity | Turtzo LC. et al., 2007 [11] |
|  | Exon-2 | c.445G>C (p.Gly149Ser) | 6m/N/A | Turkish | Hepatosplenomegaly, neutropenia, and hypofibrinogenaemia. Lymphopenia, hypertriglyceridaemia, Fever, defective NK cell activity | Trizzino A. et al., 2008 [12] |
|  | Exon-2 | c.305G>T (p.Cys102Phe) | 5y/M | China | Fever, hepatosplenomegaly, lymphadenectasis and cytopenia, EBV infection | Lu G et al., 2009 [13] |
|  | Exon-2 | c.503G>A (p.Ser168Asn) | 6y/F | China | Fever, hepatosplenomegaly,  lymphadenectasis and cytopenia | Lu G et al., 2009 [13] |
|  | Exon-2 | c.10C>T (p.Arg4Cys) | 1.4y/M | Vietnam | Hypertriglyceridemia, thrombocytopenia, hemophagocytosis, hyperferittinemia, | My LT. et al., 2009 [14] |
|  | Exon-2 | c.98G>A (p.Arg33His) | 18y/M | Asia | Fever, Hepatosplenomegaly, neutropenia, thrombocytopenia, high ferritin and triglycerides level, defective NK cell and T-cell activity | Zhang K. et al., 2011 [15] |
|  | Exon-2 | c.83G>A (p.Arg28His) | 3y/M | China | Positive EBV infection, fever, hepatosplenomegaly, neutropenia, thrombocytopenia, high ferritin and triglycerides level | Zhizhuo H. et al., 2012 [16] |
|  | Exon-2 | c.93C >G (p.Cys31Trp) | 2.5y/F | China | Fever, hepatosplenomegaly, neutropenia, thrombocytopenia, high ferritin and triglycerides level | Zhizhuo H. et al., 2012 [16] |
|  | Exon-2 | c.145G>A (p.Asp49Asn) | 14d/F | Austria | Fever, thrombocytopenia, elevated bilirubin, neutropenia, hepatosplenomegaly, raised liver enzymes, ascites, and coagulopathy | Chia J. et al., 2012 [17] |
|  | Exon-2 | c.452A>T (p.His151Leu) | 39y/M | Italy | Fever, splenomegaly, pancytopenia | Sieni et al., 2012 [18] |
|  | Exon-2 | c.90T>C (p.Cys31Gly) | 3m/M | India | Fever, hepatosplenomegaly,  CNS involvement , cytopenia, hyperferritinemia,  hypertriglyceridemia | Mhatre S. et al.,2015 [19] |
|  | Exon-2 | c.386G>C (p.Trp129Ser) | 2y/M | India | Fever, hepatosplenomegaly, anemia, neutropenia, lymphocytosis, B-cell acute lymphocytic leukemia, positive for CMV infection | Mhatre S. et al., 2014 [20] |
|  | Exon-2 | c.490C>T (p.Gln164X) | 5m/F | India | Fever, hepatosplenomegaly,  CNS involvement , cytopenia, hyperferritinemia,  hypertriglyceridemia | Mhatre S. et al,2015[19] |
|  | Exon-2 | c.528_529delinsAA (p.Cys176X) | 3m/M | India | Fever, hepatosplenomegaly,  CNS involvement, cytopenia, hyperferritinemia | Mhatre S. et al.,2015 [19] |
|  | Exon-2 | c.386G>C (p.Trp129Ser) | 3m/M | India | Fever, hepatosplenomegaly, pancytopenia, high  levels of ferritin, triglycerides, tachycardia, tachypnea, increase aPTT and PT time, hemophagocytosis in the bone-marrow | Present Study |
| *SH2D1A* | Intron-1 | c.138-3C>G  (Ex-2Del) | 6y/M | Italy | Hypogammaglobulinemia, recurrent pulmonary infection, infectious mononucleosis | Sayos J. et al., 1998 [21] |
|  | Intron-1 | c.137+2T>C | N/A | N/A | Positive EBV infection, fever, hepatomegaly, thrombocytopenia | Sumegi J. et al., 2000 [22] |
|  | Intron-1 | c.137+5G>C | 2y/M | Caucasian | Bacterial conjunctivitis, bronchopneumonia, hepatosplenomegaly, positive EBV infection | Sumegi J. et al., 2000 [22] |
|  | Intron-1 | c.138-2A>C  (Ex-2Skip) | N/A /M | N/A | Fulminant infectious mononucleosis, fever, hepatomegaly, pancytopenia | Sumegi J. et al., 2000 [22] |
|  | Intron-1 | c.137+1G>C | 7y/M | N/A | Non-Hodgkin lymphoma | Lappalainen I et al.,2000 [23] |
|  | Intron-1 | c.138-2A>G  (Ex-2Skip) | 12y/M | N/A | Hypogammaglobulinemia | Tabata Y. et al.,2005 [24] |
|  | Intron-1 | c.138-1G>A  (Ex-2Skip) | N/A | N/A | Defective NK cell and T-cell activity | Gifforf CE. et al., 2014 [25] |
|  | Intron-1 | c.138-3C>G (p.Arg47GlyfsTer34) | 1.5y/M | Indian | Fever, Hepatosplenomegaly, cytopenia, positive EBV Infection, high ferritin and triglyceride, pyrexia of unknown origin | Present Study |

* N/A: Not available, *M:Male, *F:Female, *m:months, *y:years

Referances

1. Stepp SE ,Dufourcq-Lagelouse R, Le Deist F, Bhawan S , Certain S, Mathew PA, et al. Perforin gene defect in familial hemophagocytic lymphohistiocytosis. Science. 1999;286:1957-59.
2. Ericson KG, Fadeel B, Arndor SN, Soderhall C, Samualsson A, Janka G, et al. Spectrum of Perforin Gene Mutations in Familial Hemophagocytic Lymphohistiocytosis. Am J Hum Genet. 2001;68:590–97.
3. Clementi R, zurStadt U, Savoldi G, Varoitto S, Conter V, De Fusco C, et al. Six novel mutations in the PRF1 gene in children with haemophagocytic lymphohistiocytosis. J Med Genet. 2001;38:643-46.
4. Kogawa K, Lee SM, Villanueva J, Marmer D, Sumegi J, Filipovich AH. Perforin expression in cytotoxic lymphocytes from patients with hemophagocytic lymphohistiocytosis and their family members. Blood. 2002;99:61-66.
5. Clementi R, Emmi L, Maccario R, Liotta F, Moretta L, Danesino C, et al. Adult onset and atypical presentation of hemophagocytic lymphohistiocytosis in sibling carrying PRF1mutation. Blood. 2002;100:2266-67.
6. Feldmann J, Deist F, Ouache´e-Chardin M, Certain S, Sarah A, Quartier P, et al. Functional consequences of perforin gene mutations in 22 patients with familial haemophagocytic lymphohistiocytosis. Br J Haematol. 2002;117:965–72.
7. Ueda I, Morimoto A , Inaba T, Yagi T, Hibi S, Sugimoto T, et al . Characteristic perforin gene mutations of hemophagocytic lymphohistiocytosis patients in Japan. Br J Haematol. 2003;121:503-10.
8. Al-Lamki Z, Wali YA, Pathare A, Ericson KG, Henter JI. Clinical and genetic studies of familial hemophagocytic lymphohistiocytosis in Oman: need for early treatment. Pediatr Hematol Oncol. 2003;20:603-09.
9. Molleran Lee S, Villanueva J, Sumegi J, Zhang K, Kogawa K, Davis J, et al. Characterisation of diverse PRF1 mutations leading to decreased natural killer cell activity in North American families with haemophagocytic lymphohistiocytosis. J Med Genet. 2004;41:137-44.
10. Zur Stadt, Beutel K, Kolberg K, Schneppenheim R, Kabisch H, Janka G, et al. Mutation Spectrum in Children With Primary Hemophagocytic Lymphohistiocytosis: Molecular and Functional Analyses of PRF1, UNC13D,STX11, and RAB27A. Hum Mutat. 2006;27:62-68.
11. Turtzo LC, Lin DD, Hartung H, Barker PB, Arceci R, Yohay K. A neurologic presentation of familial hemophagocytic lymphohistiocytosis which mimicked septic emboli to the brain. J Child Neurol. 2007;22:863-68.
12. Trizzino A, zur Stadt U, Ueda I, Risma K, Janka G, Ishii E, et al. Genotype-phenotype study of familial haemophagocytic lymphohistiocytosis due to perforin mutations. J Med Genet. 2008;45:15-21.
13. Lu G, Xie ZD, Shen KL, Ye LJ, Wu RH, Liu CY, et al. Mutations in the perforin gene in children with hemophagocytic lymphohistiocytosis. Chin Med J (Engl). 2009;122:2851-55.
14. My LT, Lien le B, Hsieh WC, Imamura T, Anh TN, Anh PN, et al. Comprehensive analyses and characterization of haemophagocytic lymphohistiocytosis in Vietnamese children. Br J Haematol. 2010;148:301-10.
15. Zhang K, Jordan MB, Marsh RA, Johnson JA, Kissell D, Meller J, et al. Hypomorphic mutations in PRF1, MUNC13-4, and STXBP2 are associated with adult-onset familial HLH. Blood. 2011;118:5794-98.
16. Zhizhuo H, Junmei X, Yuelin S, Qiang Q, Chunyan L, Zhengde X, et al. Screening the PRF1, UNC13D, STX11, SH2D1A, XIAP, and ITK gene mutations in Chinese children with Epstein-Barr virus-associated hemophagocytic lymphohistiocytosis. Pediatr Blood Cancer. 2012;58:410-14.
17. Chia J, Thia K, Brennan AJ, Little M, Williams B, Lopez JA, et al. Fatal immune dysregulation due to gain of glycosylation mutation in lymphocyte perforin. Blood. 2012;119:1713-16.
18. Sieni E, Cetica V, Piccin A, Gherlinzoni F, Sasso FC, Rabusin M, et al. Familial hemophagocytic lymphohistiocytosis may present during adulthood: clinical and genetic features of a small series. PLoS One. 2012;7:e44649.
19. Mhatre S, Madkaikar M, Desai M, Ghosh K. Spectrum of perforin gene mutation in familial lymphohistiocytosis (FHL) patients in India. Blood cells Mol Dis. 2015;54:250-57.
20. Mhatre S, Madkaikar M, Jijina F, Ghosh K. Unusual Clinical Presentation of Familial Hemophagocytic Lymphohistiocytosis Type-2 . J Pediatr Hematol Oncol. 2014;36:e524-27.
21. Sayos J, Wu C, Morra M, Wang N, Zhang X, Allen D, et al. The X-linked lymphoprolifertaive-disease gene product SAP regulates signal induced through the co-receptor SLAM. Nature. 1998;395:462-69.
22. Sumegi J, Huang D, Lanyi A, Davis JD, Seemayer TA, Maeda A, et al. Correlation of mutation of SH2D1A gene and Epstein-Barr virus infection with clinical phenotypes and outcome in X-linked lymphoproliferative disease. Blood. 2000;96:3118-25.
23. Lappalainen I, Giliani S, Franceschini R, Bonnefoy JY, Duckett C, Notarangelo LD, et al. Structural basis for SH2D1A mutations in X-linked lymphoproliferative disease. Biochem Biophys Res Commun. 2000;269:124-30.
24. Tabata Y, Villanueva J, Lee SM, Zhang K, Kanegane H, Miyawaki T, et al. Rapid detection of intracellular SH2D1A protein in cytotoxic lymphocytes from patients with X-linked lymphoproliferative disease and their family members. Blood. 2005;105:3066-71.
25. Gifford CE, Weingartner E, Villanueva J, Johnson J, Zhang K, Filipovich AH, et al. Clinical flow cytometric screening of SAP and XIAP expression accurately identifies patients with SH2D1A and XIAP/BIRC4 mutations. Cytometry B Clin Cytom. 2014;86:263-71.
